# Development and external validation of a machine learning model for predicting in-hospital mortality in ICU patients with diabetic kidney disease: a study utilizing the MIMIC database and a Chinese cohort

**DOI:** 10.3389/fendo.2026.1699647

**Published:** 2026-02-27

**Authors:** YuNan Han, RuMeng Mao, ChengYue Xiong, YongXiang Wang, Lin Li, HongLian Wang

**Affiliations:** 1 Department of Endocrinology, The First Affiliated Hospital of Yangtze University, Jingzhou, Hubei, China; 2 Department of Medicine, Yangtze University, Jingzhou, Hubei, China

**Keywords:** all-cause mortality, diabetes nephropathy, machine learning, MIMIC-IV, SHAP

## Abstract

**Background:**

Patients with diabetic kidney disease (DKD) admitted to the intensive care unit (ICU) face an exceptionally high risk of in-hospital mortality. Currently, effective tools for their early risk stratification are critically lacking. Therefore, this study aimed to develop and externally validate an interpretable machine learning (ML) model for predicting in-hospital mortality in this high-risk ICU-DKD patient population.

**Methods:**

This retrospective cohort study involved developing and evaluating eight ML algorithms. Model performance was rigorously assessed using receiver operating characteristic (ROC) curves, calibration curves, and decision curve analysis (DCA). SHapley Additive exPlanations (SHAP) provided model interpretability. Data from DKD patients with ≥24-hour ICU stays were extracted from the MIMIC-IV database (n=3,403) for model development. An independent external validation cohort (n=260) was collected from the First Affiliated Hospital of Yangtze University (YTU-ICU). The primary outcome was in-hospital mortality. Lasso regression identified key predictors. Model evaluation focused on the area under the ROC curve (AUROC), calibration, and net clinical benefit.

**Results:**

Ten features were selected for model development. Among the tested algorithms, XGBoost demonstrated superior predictive performance, achieving an AUROC of 0.738 (internal validation) and 0.746 (external validation), with corresponding accuracies of 72.18% and 72.69%. SHAP analysis highlighted respiratory failure, lymphocyte count, SOFA score, RDW, age, and SAPS II as the six most important predictors.

**Conclusions:**

The developed XGBoost model demonstrates good predictive performance for in-hospital mortality in ICU-DKD patients, exhibiting satisfactory generalizability and interpretability. This tool supports early risk stratification and facilitates personalized treatment strategies in critical care settings.

## 1 Introduction

Diabetes mellitus (DM), a major medical concern of this century, continues to pose a substantial threat to human health. The Global Burden of Diseases, Injuries, and Risk Factors Study (GBD) 2019 estimates that diabetes ranked as the eighth most significant contributor to death and disability, affecting nearly 460 million people of all ages and in all countries worldwide in 2019 (1). Diabetic kidney disease (DKD), a prevalent complication of diabetes, is identified by a prior diagnosis of diabetes, indicators of kidney damage (such as a reduced glomerular filtration rate [GFR]) and ongoing albuminuria (2) As DM becomes more common, the incidence of DKD is also on the rise (3, 4). DKD now represents a major etiology of end-stage renal disease (ESRD) (5), resulting in diminished quality of life, reduced survival, and a significant socioeconomic burden (6). Patients with DKD have been shown to have a significantly increased risk of all-cause, in-hospital mortality (7, 8). Moreover, the admission rate to the intensive care unit (ICU) for patients with ESRD, including those with ESRD secondary to DKD, is reported to be between 1% and 9%. This rate is substantially higher than that observed in the general population (9). In light of the accelerated progression and marked clinical heterogeneity characteristic of DKD, the development of reliable and effective prognostic models is essential. DKD is fundamentally a complex pathological process involving multiple systems. Patients with DKD frequently exhibit concomitant cardiovascular dysfunction, electrolyte disturbances, and multiple metabolic abnormalities (10). These dynamic physiological parameters often demonstrate complex, nonlinear interactions. Traditional prediction approaches, like logistic regression (LR) models, have been extensively utilized for clinical risk prediction. Nevertheless, the underlying linear assumption of these models often fails to adequately capture the synergistic or antagonistic effects between multidimensional biomarkers. For example, high glucose levels can exacerbate glomerular hypertension by activating the renin-angiotensin-aldosterone system (RAAS). Concurrent hypoalbuminemia further exacerbates this pathophysiology through disturbances of the Starling forces (11–13). The linear combination of variables in LR models limits their ability to capture these multi-factorial, non-linear interactions. Furthermore, Jin et al. (14) proposed a predictive model to estimate 1-year mortality across individuals with severe DKD. However, its reliance on conventional Cox regression and the absence of external validation limit its generalizability.

In contrast to the limitations inherent in traditional statistical modeling, machine learning (ML) techniques offer novel approaches to predicting complex clinical outcomes. A key advantage of ML algorithms lies in their capacity to elucidate non-linear relationships and interaction effects among high-dimensional variables via adaptive learning, without relying on pre-specified assumptions (15). In recent years, ML has shown broad application prospects for forecasting the outcomes in critically ill patients. Fan et al. (16) built and assessed an ML model designed to forecast the outcomes of critically ill individuals experiencing sepsis-associated acute kidney injury (SA-AKI). Risk factors were then assessed and the correlation of these factors with patient outcomes was quantitatively visualized utilizing the SHapley Additive exPlanations (SHAP) approach. Wang et al. (17) developed nine ML algorithms for predictive modeling, ultimately selecting the optimal model to forecast in-hospital mortality in critically ill AKI patients receiving continuous renal replacement therapy (CRRT). The model was then visualized as both static bar charts and web-based dynamic bar charts for clinical application. Xie et al. (18) employed XGBoost and LR to identify risk factors and subsequently construct an ML model and nomogram for predicting in-hospital mortality in patients with severe diabetic ketoacidosis. The model demonstrated robust predictive performance in both the MIMIC-IV and eICU data banks. According to aforementioned research findings, ML algorithms can advance the creation and verification of predictive models within critical care explorations, leading to more reliable and accurate patient prognosis predictions. However, the existing literature has primarily focused on specific diseases or treatment modalities and has not yet explored the application to the specific population with critically ill DKD. Furthermore, the prediction time points differ among most studies, and the absence of adequate external validation in some models constrains their generalizability and applicability to different patient groups.

A primary goal of this research was to build and test a comprehensive ML model to estimate the in-hospital death from any cause across individuals with DKD utilizing the MIMIC-IV database. To ensure the model’s broad usability across various healthcare settings, external validation will be conducted to evaluate its generalizability. The findings of this study will provide a scientific basis for clinical practice, supporting early risk stratification and personalized interventions for patients with DKD. Furthermore, the findings will offer a theoretical foundation and technical guidance for the creation of Clinical Decision Support Systems (CDSS), thereby facilitating the application of data-driven precision medicine in the management of DKD.

## 2 Methods

### 2.1 Ethics and informed consent statement

The MIMIC-IV database was de-identified to protect patient privacy. Therefore, the Institutional Review Board (IRB) of Beth Israel Deaconess Medical Center waived the requirement for informed consent from patients. This research was reported according to the Reporting of Studies Conducted utilizing Observational Routinely collected Health Data guidelines (19), the Declaration of Helsinki, and the NIH “Protecting Human Research Participants” (PHRP) online course.

The YTU Ethics Committee reviewed and approved this research (Approval No.: KY2024-087-01). Since we employed retrospective research with anonymized data that cannot identify individual patients, informed consent was not required. Rigorous adherence to all privacy rights of human subjects was maintained. All data were anonymized to protect personal information and ensure confidentiality.

### 2.2 Description of data sources

This retrospective research adopted health-correlated information sourced from the MIMIC-IV (V3.1), a large-scale and publicly accessible resource created and maintained by the Laboratory for Computational Physiology at MIT. The resource consists of a large collection of detailed health records obtained from individuals admitted to ICU at Beth Israel Deaconess Medical Center (20). To meet database access requirements, one author (YuNan Han) completed the National Institutes of Health’s web-based course “Protecting Human Research Participants” (Record ID: 65,640,325) and was responsible for extracting the data. Additionally, data from ICU-DKD individuals at the First Affiliated Hospital of Yangtze University were gathered for external verification purposes, forming the YTU-ICU database.

### 2.3 Study population

Individuals confirmed with DKD, based on the International Classification of Diseases, Ninth (ICD-9) (250.40, 250.41, 250.42, and 250.43) and Tenth Revision (ICD-10) (E082, E0821, E0822, E0829; E102, E1021, E1022, E1029; E112, E1121, E1122, E1129; E132, E1321, E1322, E1329), were enrolled into the research. Patients were included if they met the following criteria: (1) a stay in the ICU of at least 24 hours; (2) complete data for categorical variables such as gender, marital status, comorbidities, medication history, and medical procedures. Only data from the first hospital stay were gathered if the patient had been admitted more than once. Data was gathered on clinical variables during the initial 24 hours following admission to the ICU. The primary outcome was in-hospital mortality, defined as death from any cause occurring at any time point during the hospital stay, regardless of the duration of the ICU or hospital stay. While predictors were restricted to the first 24 hours of ICU admission to facilitate early risk stratification, the outcome was recorded for the entire hospitalization period.

### 2.4 Data collection

Information was extracted utilizing Navicate Premium (V17) by executing Structured Query Language (SQL) queries. The extraction of potential variables was divided into five primary categories: (1) Demographic information collected, encompassing age, gender, marital status, weight, height, and body mass index (BMI). (2) Comorbidities, such as acidosis, arrhythmias, electrolyte imbalances, cardiovascular diseases, pneumonia, respiratory failure, and sepsis. (3) Laboratory markers, including Albumin, ALT, AST, APTT, Bicarbonate, BUN, Chlorine, Creatinine, Glucose, Hemoglobin, Lymphocyte, Neutrophils, Platelets, Potassium, red cell distribution width (RDW), Prothrombin time, Sodium, Total bilirubin, Leucocyte. (4) Severity of illness scores at admission, involving the Simplified Acute Physiology Score II (SAPS-II), the Sepsis-correlated Organ Failure Assessment Score (SOFA) (21, 22), and the Glasgow Coma Scale (GCS), were collected. (5) Frequently administered ICU medications, including insulin, cephalosporins, omeprazole, dexamethasone, meropenem, and vancomycin.

### 2.5 Data preprocessing and feature selection

To decrease potential biases, any variable with over 20% missing values was ruled out from this research. For variables with data missing less than 20%, the “mice” package in R software was used for multiple imputation (23). Outliers in the data were managed using the Winsorization method to minimize the impact of extreme values on the analysis results (24). The MIMIC-IV data bank was subjected to random partitioning into a training set (70%) and an internal validation set (30%). A lasso logistic regression model was implemented solely on the training set to select the most relevant predictors, and the selected features were then applied to the internal validation and external validation sets. This rigorous approach was adopted to rule out data leakage and ensure the integrity of the internal validation. The lambda parameter was determined utilizing ten-fold cross-validation within the training set. For the external validation cohort (YTU-ICU), complete-case analysis was employed because the proportion of missing data among the selected predictors was negligible (less than 1%) (**Supplementary Table S1**). Therefore, no imputation models were applied to the external set.

### 2.6 Model development and interpretation

Eight ML algorithms were employed for forecasting the in-hospital death from any cause risk among individuals with DKD. These algorithms encompassed Extreme Gradient Boosting (XGBoost), gradient boosting decision trees (GBDT), light gradient boosting machine (LightGBM), neural networks (NN), LR, naive Bayes (NB), random forest (RF), and support vector machine (SVM). To achieve optimal predictive performance, hyperparameter tuning was conducted for complex non-linear algorithms, including XGBoost, GBDT, LightGBM, RF, SVM, and NN, using a grid search strategy. During this process, models were trained based on the training set, and model performance was evaluated based on the internal validation set to identify the best hyperparameter combinations. Default parameters were used for logistic regression and NB. Evaluation metrics included the area under the receiver operating characteristic curve (AUROC), accuracy, Youden’s index, sensitivity, specificity, F1-score, positive predictive value (PPV), and negative predictive value (NPV). Net clinical benefit was assessed through decision curve analysis (25). Calibration curves were employed to assess the model’s reliability. The optimal model was defined as the model with the most robust balance of discrimination, calibration, and clinical utility within the internal validation set. To assess the model’s adaptability and generalizability across different contexts, its external validity was tested utilizing an independent data bank from the YTU-ICU database. SHAP summary plots were generated to illustrate the role of each feature on the predictive outcome. Additionally, SHAP analysis demonstrated the individual-level contribution of each feature within specific samples through individual case assessments using force plots, thereby facilitating a deeper understanding of the model’s decision-making process.

To further ensure reproducibility and minimize the risk of overfitting, a fixed random seed (123) was utilized for all procedures. Regarding class imbalance, we opted to train all models on the original data distribution to reflect real-world clinical prevalence (~18.2%), utilizing AUROC and F1-score as robust evaluation metrics. Performance was estimated using 95% confidence intervals (CIs) calculated via 2000 bootstrap replicates. Comprehensive details regarding the specific R packages used, hyperparameter tuning ranges, and final model configurations are summarized in **Supplementary Table S2**.

### 2.7 Statistical methods

Data normality was assessed utilizing the Shapiro-Wilk test. Normally distributed continuous data were summarized utilizing means (SD, standard deviation) and were compared utilizing independent samples t-tests. The Mann-Whitney U test was employed to compare variables that were not normally distributed, with data summarized utilizing medians and interquartile ranges (IQRs). The chi-squared test or Fisher’s exact test was employed to compare categorical variables, with data summarized utilizing frequencies and percentages. R (V4.4.1) was adopted for data analysis and all computations. The criterion for statistical significance was P < 0.05 for a two-tailed hypothesis.

Given the outcome imbalance and differences in event rates between the development and external cohorts, we evaluated the discrimination of the model using the AUROC and the area under the precision–recall curve (PR-AUC). Calibration was quantified using the Brier score, calibration-in-the-large (calibration intercept), and the calibration slope by regressing observed outcomes on the logit of predicted probabilities. Clinical utility was assessed using decision curve analysis by estimating net benefit across threshold probabilities from 0.1 to 0.5. Uncertainty for all metrics was estimated using nonparametric bootstrap resampling (2,000 iterations), and results were reported as point estimates with 95% CIs.

## 3 Results

### 3.1 Population demographics

Initially, 10,686 patients with DKD were screened. Following the application of inclusive and exclusive criteria, 3,403 cases were ultimately enrolled in the final investigation. Concurrently, 261 patients from YTU were screened, with 260 ultimately retained as an external validation cohort, as detailed in **Supplementary Figure S1**. Detailed information on the differences between the survival and mortality groups within the MIMIC-IV database can be found in **Table 1**. Marked differences were observed between the two groups in terms of age, systolic blood pressure, pulse, respiratory rate, ALB, ALT, AST, APTT, BUN, CL, CR, HB, LYM, NEU, PT, RDW, TB, Acidosis, Arrhythmia, Electrolyte disturbance, Pneumonia, Respiratory failure, Sepsis, Cephalosporin, Dexamethasone, Meropenem, Vancomycin, CRRT, MV, GCS, SAPSII, and SOFA.

**Table 1:** All predictor variables for critically Ill patients with DKD in the MIMIC-IV database collection.

Characteristics	Overall (N = 3,403)	Alive (N = 2,783)	Death (N = 620)	P value
Demographics				
Gender, n(%)				0.385
Male	2,124 (62.4%)	1,747 (62.8%)	377 (60.8%)	
Female	1,279 (37.6%)	1,036 (37.2%)	243 (39.2%)	
Age (years)	67.0 [58.0;75.0]	67.0 [58.0;75.0]	69.0 [61.0;76.2]	<0.001
Marital status, n(%)				0.97
Single	962 (28.3%)	785 (28.2%)	177 (28.5%)	
Divorced/Widowed	620 (18.2%)	506 (18.2%)	114 (18.4%)	
Married	1,821 (53.5%)	1,492 (53.6%)	329 (53.1%)	
BMI	30.4 [26.4;35.5]	30.4 [26.5;35.5]	29.9 [25.8;35.4]	0.171
Vital signs				
DBP (mmHg)	132 [120;146]	132 [120;147]	130 [119;146]	0.078
SBP (mmHg)	72.0 [63.0;80.0]	72.0 [63.0;80.0]	70.0 [61.0;80.0]	0.019
Pulse (bpm)	98.0 [96.0;100]	98.0 [96.0;100]	98.0 [95.0;100]	0.005
Respiratory (breaths/min)	18.0 [15.0;22.0]	18.0 [15.0;22.0]	19.0 [16.0;23.0]	<0.001
Laboratory indicators				
ALB (g/dL)	3.40 [2.90;3.90]	3.40 [3.00;3.90]	3.20 [2.70;3.70]	<0.001
ALT (U/L)	21.0 [13.0;38.0]	20.0 [13.0;36.0]	23.0 [14.0;47.0]	0.002
APTT (sec)	30.7 [27.3;36.5]	30.4 [27.1;35.8]	32.3 [28.0;40.2]	<0.001
AST (U/L)	27.0 [19.0;50.0]	26.0 [18.0;47.0]	32.5 [20.0;70.2]	<0.001
BIC (mmol/L)	22.0 [19.0;25.0]	22.0 [19.0;25.0]	22.0 [18.0;25.0]	0.107
BUN (mg/dL)	30.0 [20.0;49.0]	29.0 [19.0;46.0]	38.0 [24.0;62.0]	<0.001
CL (mmol/L)	103 [99.0;107]	104 [99.0;107]	101 [97.0;106]	<0.001
CR (mg/dL)	1.60 [1.10;2.70]	1.50 [1.10;2.50]	1.90 [1.30;3.40]	<0.001
GLU (mg/dL)	152 [117;211]	150 [117;211]	157 [115;210]	0.368
HB (g/dL)	9.60 [8.20;11.3]	9.70 [8.20;11.3]	9.40 [8.10;11.1]	0.049
LYM (10^9^/L)	1.25 [0.78;1.84]	1.30 [0.83;1.87]	1.04 [0.60;1.59]	<0.001
NEU (10^9^/L)	6.61 [4.53;10.1]	6.43 [4.48;9.75]	7.62 [4.78;11.2]	<0.001
PLT (10^9^/L)	181 [132;241]	181 [134;242]	179 [127;239]	0.26
K (mmol/L)	4.40 [3.90;4.90]	4.40 [3.90;4.80]	4.40 [3.90;4.90]	0.872
PT (sec)	13.9 [12.3;16.6]	13.8 [12.2;16.4]	14.5 [12.8;18.2]	<0.001
RDW (%)	14.7 [13.7;16.3]	14.6 [13.6;16.1]	15.4 [14.3;17.1]	<0.001
NA (mmol/L)	138 [135;141]	138 [135;141]	138 [134;141]	0.269
TB (mg/dL)	0.50 [0.30;0.80]	0.50 [0.30;0.80]	0.50 [0.30;0.90]	<0.001
LEU (10^9^/L)	10.2 [7.40;14.1]	10.1 [7.40;14.0]	10.9 [7.50;14.6]	0.109
Comorbidity disease, n(%)				
Acidosis:				<0.001
No	2,031 (59.7%)	1,786 (64.2%)	245 (39.5%)	
Yes	1,372 (40.3%)	997 (35.8%)	375 (60.5%)	
Arrhythmia:				<0.001
No	3,087 (90.7%)	2,548 (91.6%)	539 (86.9%)	
Yes	316 (9.29%)	235 (8.44%)	81 (13.1%)	
CVD:				0.405
No	1,188 (34.9%)	981 (35.2%)	207 (33.4%)	
Yes	2,215 (65.1%)	1,802 (64.8%)	413 (66.6%)	
Electrolyte disturbance:				0.029
No	3,334 (98.0%)	2,734 (98.2%)	600 (96.8%)	
Yes	69 (2.03%)	49 (1.76%)	20 (3.23%)	
Pneumonia:				<0.001
No	2,592 (76.2%)	2,192 (78.8%)	400 (64.5%)	
Yes	811 (23.8%)	591 (21.2%)	220 (35.5%)	
Respiratory failure:				<0.001
No	2,073 (60.9%)	1,846 (66.3%)	227 (36.6%)	
Yes	1,330 (39.1%)	937 (33.7%)	393 (63.4%)	
Sepsis:				0.001
No	3277 (96.3%)	2694 (96.8%)	583 (94.0%)	
Yes	126 (3.70%)	89 (3.20%)	37 (5.97%)	
Medication history				
Cephalosporin, n(%)				<0.001
No	1,402 (41.2%)	1,220 (43.8%)	182 (29.4%)	
Yes	2,001 (58.8%)	1,563 (56.2%)	438 (70.6%)	
Dexamethasone:				0.04
No	3,264 (95.9%)	2,679 (96.3%)	585 (94.4%)	
Yes	139 (4.08%)	104 (3.74%)	35 (5.65%)	
Insulin:				0.655
No	1,132 (33.3%)	931 (33.5%)	201 (32.4%)	
Yes	2,271 (66.7%)	1,852 (66.5%)	419 (67.6%)	
Meropenem:				<0.001
No	3,191 (93.8%)	2,650 (95.2%)	541 (87.3%)	
Yes	212 (6.23%)	133 (4.78%)	79 (12.7%)	
Omeprazole:				0.146
No	2,794 (82.1%)	2,298 (82.6%)	496 (80.0%)	
Yes	609 (17.9%)	485 (17.4%)	124 (20.0%)	
Vancomycin:				<0.001
No	1,731 (50.9%)	1,523 (54.7%)	208 (33.5%)	
Yes	1,672 (49.1%)	1,260 (45.3%)	412 (66.5%)	
Medical operation, n(%)				
CRRT:				<0.001
No	3,274 (96.2%)	2,712 (97.4%)	562 (90.6%)	
Yes	129 (3.79%)	71 (2.55%)	58 (9.35%)	
MV:				<0.001
No	1,892 (55.6%)	1,596 (57.3%)	296 (47.7%)	
Yes	1,511 (44.4%)	1,187 (42.7%)	324 (52.3%)	
Medical scores				
GCS	15.0 [15.0;15.0]	15.0 [15.0;15.0]	15.0 [15.0;15.0]	0.001
SAPSII	38.0 [30.0;46.0]	37.0 [29.0;45.0]	41.5 [33.0;52.0]	<0.001
SOFA	5.00 [3.00;7.00]	5.00 [3.00;7.00]	7.00 [4.00;9.00]	<0.001

BMI, Body Mass Index; DBP, Diastolic Blood Pressure; SBP, Systolic Blood Pressure; Alb, Albumin; ALT, Alanine Aminotransferase; APTT, Activated Partial Thromboplastin Time; AST, Aspartate Aminotransferase; Bic, Bicarbonate; BUN, Blood Urea Nitrogen; CL, Chloride; Cr, Creatinine; GLU, Glucose; HB, Hemoglobin; Lym, Lymphocyte; NEU, Neutrophile; PLT, Platelet; K, Potassium; PT, Prothrombin Time; RDW, Red Cell Distribution Width; NA, Sodium; TB, Total Billrubin; LEU, Leukocyte; CVD, Cardiovascular Disease; CRRT, Continuous Renal Replacement Therapy; MV, Mechanical Ventilation; GCS, Glasgow Coma Scale; SAPSII, Simplified Acute Physiology Score II; SOFA, Sequential Organ Failure Assessment.

### 3.2 Feature selection

The screening procedure utilized forty-five clinical features initially collected as possible input variables. To pinpoint significant variables linked to the risk of death from any cause among individuals with DKD, LASSO regression was utilized. The tuning parameter λ, which controlled the penalty on the β coefficients (shrinkage parameter, λ = 0.026; lambda.1se; **Supplementary Figure S2**), was applied to regulate coefficient shrinkage. Ten underlying predictors whose coefficients were non-zero were identified, specifically age, lymphocyte count, RDW, acidosis, pneumonia, respiratory failure, meropenem, vancomycin, SAPS II, and SOFA score. Model creation was then conducted utilizing these predictors.

### 3.3 Model assessment and comparison

**Figure 1:**
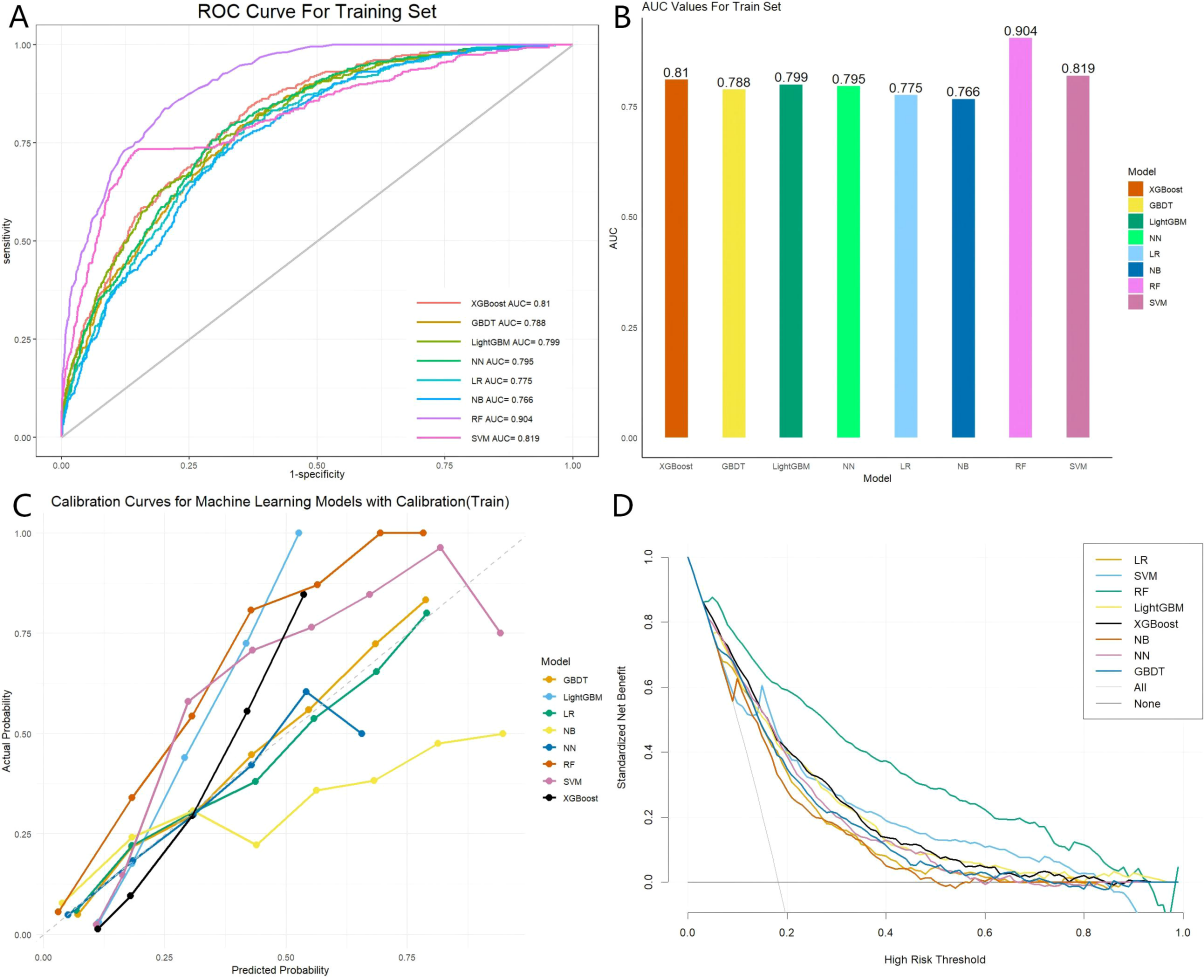
Performance evaluation of eight models within the training set. **(A)** Receiver operating characteristic (ROC) curves for each model; **(B)** Area under the curve (AUC) values for each model; **(C)** Calibration curves for each model; **(D)** Standardized net benefit analysis for each model. The models included are: XGBoost (Extreme Gradient Boosting), GBDT (Gradient Boosting Decision Tree), LightGBM (Light Gradient Boosting Machine), NN (Neural Network), LR (Logistic Regression), NB (Naive Bayes), RF (Random Forest), and SVM (Support Vector Machine).

**Figure 2:**
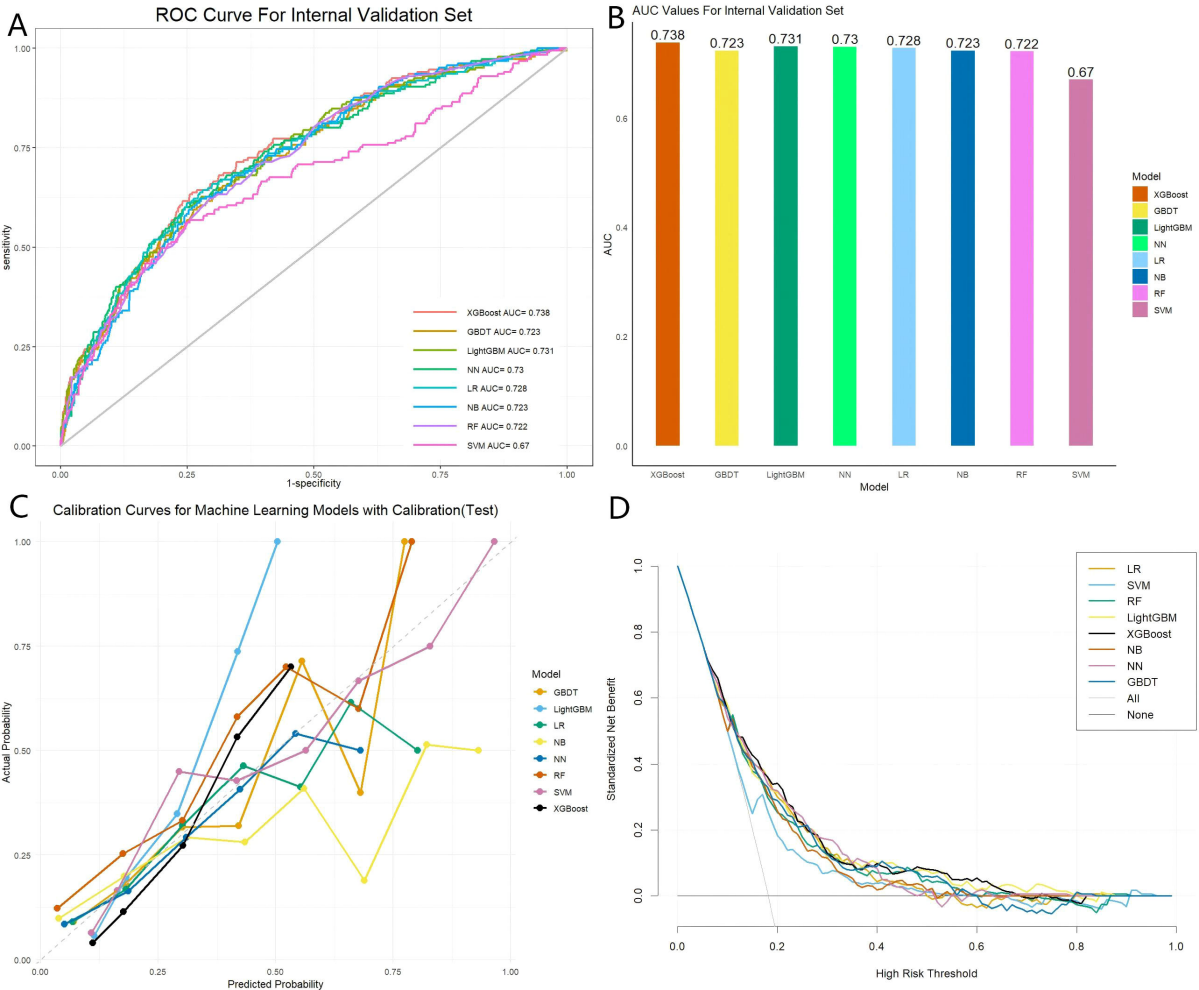
Performance evaluation of eight models within the internal validation set. **(A)** Receiver operating characteristic (ROC) curves for each model; **(B)** Area under the curve (AUC) values for each model; **(C)** Calibration curves for each model; **(D)** Standardized net benefit analysis for each model. The models included are: XGBoost (Extreme Gradient Boosting), GBDT (Gradient Boosting Decision Tree), LightGBM (Light Gradient Boosting Machine), NN (Neural Network), LR (Logistic Regression), NB (Naive Bayes), RF (Random Forest), and SVM (Support Vector Machine).

The training data bank was adopted to build eight distinct ML models, specifically: XGBoost, GBDT, LightGBM, NN, LR, NB, RF, and SVM. The capacity of each model was examined on the training and internal validation data banks utilizing a comprehensive suite of metrics: AUROC, accuracy, optimal cutoff point, Youden’s index, specificity, sensitivity, F1-score, PPV, and NPV. **Figures 1A, B**, **2A, B** display the ROC curves and corresponding AUROC scores for each model, based on their capacity on the training and internal validation data banks. In the internal validation data bank, XGBoost demonstrated the highest AUROC of 0.738, followed by LightGBM (0.731), NN (0.73), LR (0.728), GBDT and NB (0.723), RF (0.722), and SVM (0.67). **Supplementary Table S3** and **Supplementary Figure S3** provide a comprehensive overview of the models’ predictive capabilities. **Figures 1C**, **2C** depict the calibration curves for each model, showing their capacity on the training and internal validation sets, respectively. Overall, the XGBoost model demonstrated the best calibration performance across both datasets. DCA was employed to explore the net clinical benefit of each model across a range of threshold probabilities, as illustrated in **Figures 1D**, **2D**. The “treat-all” approach assumes positive for every case, which may result in unnecessary treatment and inefficient use of resources. Conversely, the “no-treatment” strategy assumes that all cases are negative, which could lead to inadequate treatment and heightened risks for patients. As illustrated in **Figures 2D**, the net benefit of the XGBoost model surpasses both the “treat-all” and “treat-none” strategies over a broad spectrum of threshold probabilities. This finding underscores the clinical value of the model in effectively estimating the risk of death from any cause and minimizing inappropriate treatment - both overtreatment and undertreatment - for patients.

Furthermore, to mitigate the risk of overfitting, the LASSO 1-standard-error rule was used to select parsimonious features, and regularization was used for non-linear algorithms. The AUROC (0.738) in internal validation was similar to the AUROC (0.746) in the external validation, suggesting that model discrimination transferred reasonably well and performance was unlikely to be influenced by overfitting to the training data. Nevertheless, as calibration is known to be cohort-dependent, local recalibration and threshold adaptation may still be required before clinical deployment.

In addition to AUROC and accuracy, we estimated PR-AUC, calibration metrics, and net benefit with bootstrap uncertainty estimates. Overall, discrimination and PR-AUC were consistent between the training and internal validation cohorts. For the primary model (XGBoost), AUROC was 0.810 (95% CI 0.790–0.830) in the training set and 0.738 (0.697–0.775) in the internal validation set, with corresponding PR-AUC values of 0.497 (0.449–0.544) and 0.411 (0.342–0.486), respectively. Calibration-in-the-large was generally close to 0 in the training cohort, whereas calibration slopes often deviated from 1, suggesting over-dispersion in some models. Decision curve analysis showed positive net benefits across clinically relevant thresholds (**Supplementary Tables S4**, **S5**).

### 3.4 External validation

**Figure 3:**
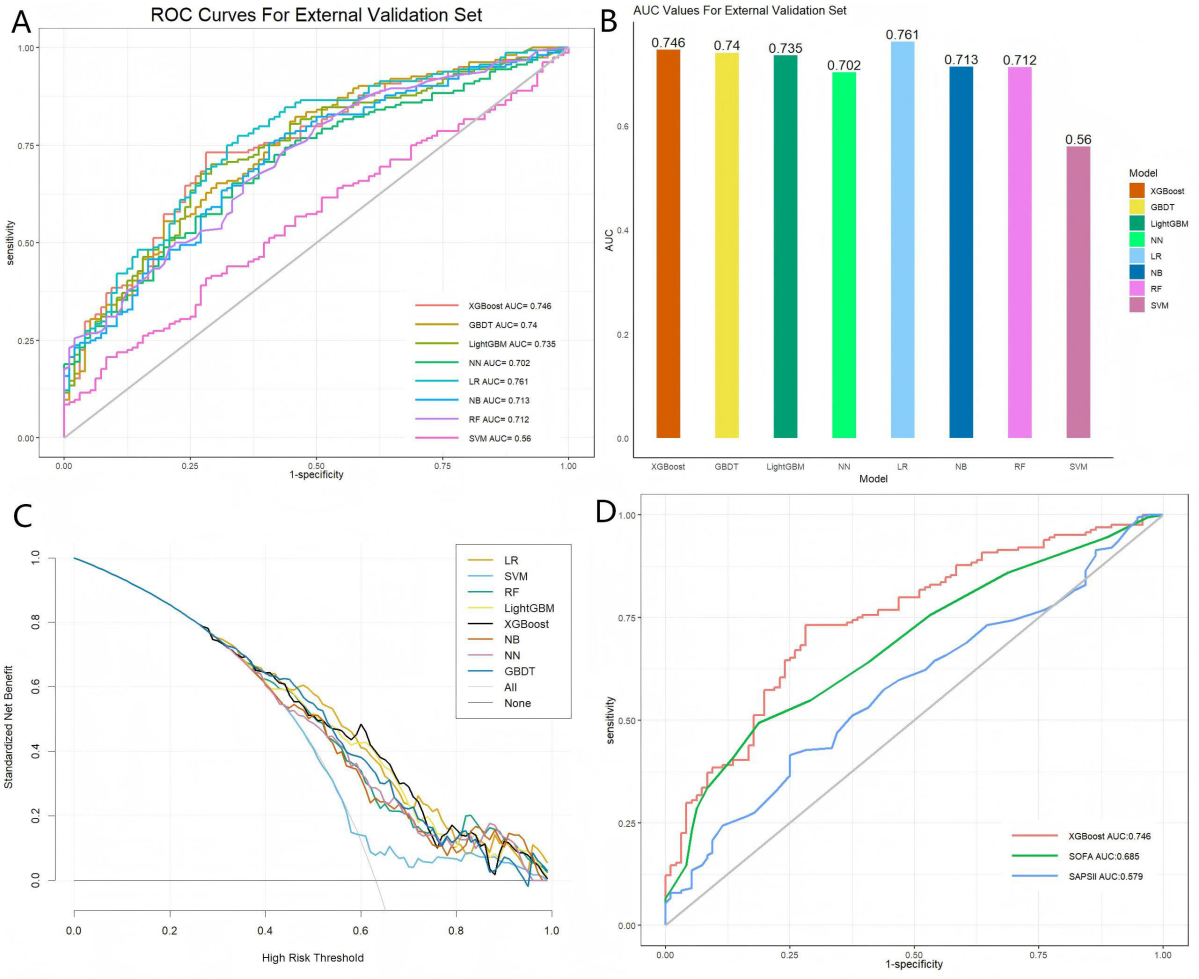
Performance evaluation of eight models within the external validation set, along with a comparison to the optimal and traditional models. **(A)** Receiver operating characteristic (ROC) curves for each model; **(B)** Area under the curve (AUC) values for each model; **(C)** Standardized net benefit analysis for each model; **(D)** Comparison of the XGBoost model with the SOFA and SAPSII scores. The models included are: XGBoost (Extreme Gradient Boosting), GBDT (Gradient Boosting Decision Tree), LightGBM (Light Gradient Boosting Machine), NN (Neural Network), LR (Logistic Regression), NB (Naive Bayes), RF (Random Forest), and SVM (Support Vector Machine). SOFA (Sequential Organ Failure Assessment) and SAPSII (Simplified Acute Physiology Score II) are also considered for comparison.

The YTU-ICU database, containing data from 260 patients, was employed to further validate the predictive performance of each model for death from any cause across DKD patients. As illustrated in **Supplementary Table S6**, the external validation set consisted of patients with a higher average age in comparison to the training cohort. Furthermore, the external validation set exhibited an elevated proportion of deceased patients in comparison to survivors. In comparison to the training set, individuals in the external validation cohort exhibited lighter body weights, increased proportions of arrhythmias, respiratory failure, and sepsis, and higher SAPSII scores. **Figures 3A, B** illustrate the ROC curves and AUROC values for each model within the external validation set. The LR model exhibited superior capacity in the external validation cohort, achieving an AUROC of 0.761. The AUROC scores for the remaining models were: XGBoost (0.746), GBDT (0.74), LightGBM (0.735), NB (0.731), RF (0.712), NN (0.702), and SVM (0.56). The predictive performance of these models is detailed in **Supplementary Table S7**. In the external validation cohort, although the LR model achieved a slightly higher AUROC (0.761) than XGBoost (0.746), the XGBoost model was ultimately selected as the optimal model due to its superior clinical utility. As demonstrated in the DCA (**Figure 3C**), XGBoost provided a consistently higher net clinical benefit compared to LR and other models across a wide range of threshold probabilities (0.1–0.6). This result indicated that XGBoost was more effective in guiding clinical interventions and reducing unnecessary harm in a real-world ICU setting. **Figure 3D** illustrates the superior predictive performance of the XGBoost model relative to conventional scoring systems such as SOFA and SAPSII. However, the calibration curve exhibited poor fit in the external validation cohort, which may be attributable to the small sample size of the YTU-ICU dataset and variations in patient demographics relative to MIMIC-IV.

In the external validation cohort, the primary model maintained comparable discrimination, with an AUROC of 0.746 (95% CI 0.686–0.805) and PR-AUC of 0.834 (0.774–0.887). However, calibration was substantially degraded, as reflected by a markedly positive calibration intercept (1.783, 95% CI 1.532–2.043) and a calibration slope greater than 1 (1.954, 1.422–2.647), indicating systematic miscalibration under different baseline risks. These findings suggested that while discrimination transferred reasonably well across cohorts, local recalibration and cohort-specific threshold adaptation may be required before clinical deployment. Decision curve analysis demonstrated net benefits across clinically relevant thresholds (**Supplementary Tables S4, S5**).

### 3.5 Interpretability analysis

**Figure 4:**
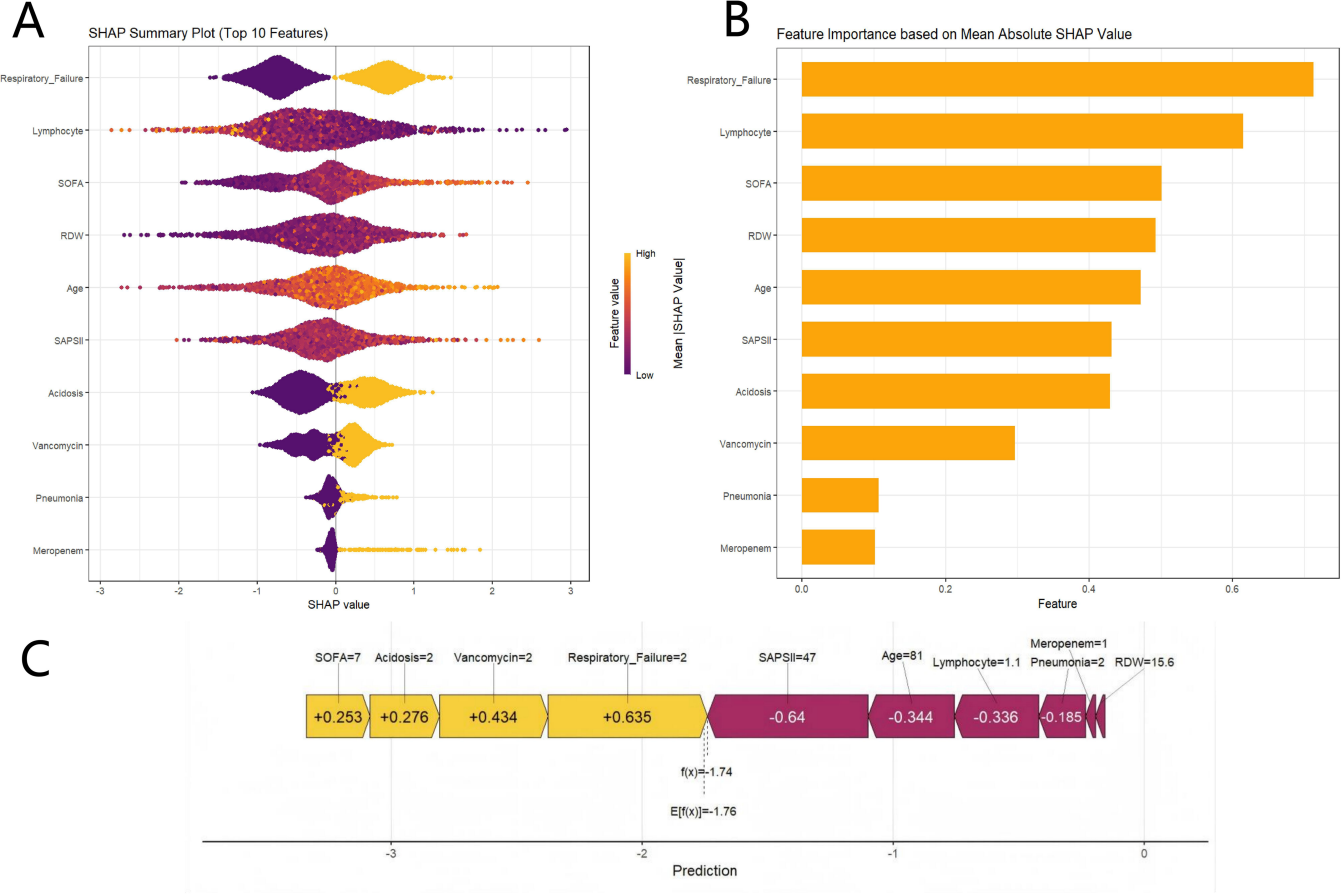
SHAP-based interpretation of the model. **(A)** Explanation of the 10 clinical features predicting mortality using the XGBoost model. **(B)** Ranking of the importance of the 10 clinical features in XGBoost based on the average absolute SHAP values (|SHAP values|). **(C)** SHAP force plot for a single patient.

**Figure 5:**
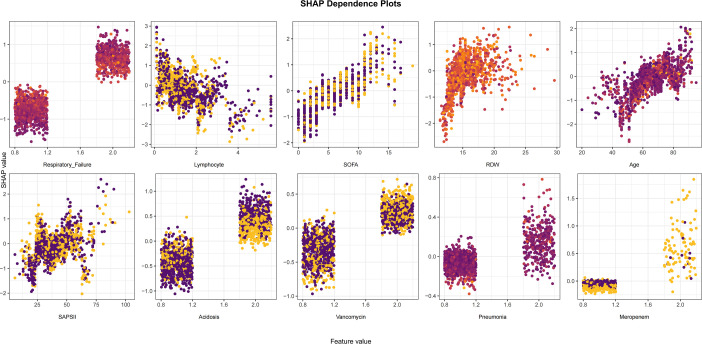
Partial dependence plot of the XGBoost model based on SHAP.

**Figure 4A** displays a comprehensive swarm plot for the XGBoost model. The x-axis indicates SHAP values, and the y-axis shows the features, ordered according to their impact as determined by cumulative SHAP values. Each data point represents a specific individual case; the x-axis of the point reflects the SHAP value of that instance for the specified feature. Respiratory failure, Lymphocyte, SOFA, RDW, Age, SAPS II, Acidosis, Vancomycin, Pneumonia, and Meropenem were identified as the 10 most important factors, ranked in order of importance, for forecasting the risk of death from any cause in individuals with DKD (**Figure 4B**). Respiratory failure, acidosis, pneumonia, vancomycin administration, and meropenem administration were identified as features characteristic of mortality and exhibited positive SHAP values, thus contributing to a model prediction favoring mortality. Lymphocyte count, SOFA score, RDW, age, and SAPS II score further contributed to the prediction of mortality. SHAP dependency plots were employed to further analyze the influence of 10 factors on the mortality risk prediction by the XGBoost model. As shown in **Figure 5**, respiratory failure, acidosis, severe pneumonia, and the use of vancomycin and meropenem were significantly associated with elevated mortality. Notably, the mortality risk increased with the severity of respiratory failure and acidosis. Conversely, a decreased lymphocyte count has a correlation with an elevated risk of death, whereas a higher lymphocyte count might reduce the risk of mortality. Higher SOFA scores, older age, elevated RDW, and higher SAPS II scores showed a close correlation with an elevated risk of death, with these factors contributing more markedly to the risk of mortality at higher values. In addition, increased use of vancomycin and meropenem was correlated significantly with elevated risks of mortality, indicating that the administration of these antibiotics may serve as a clinical proxy for patients with a more critical status and severe or resistant infections. **Figure 4C** presents a detailed case study, illustrating how the model generates a prediction for an individual patient. In this visualization, yellow indicators denote factors that positively contribute to the prediction, whereas purple indicators represent factors that negatively impact the prediction. The baseline value, E[f(*x*)], represents the average prediction of the model when no feature information is considered. In this case, it is -1.76. The predicted value for a specific sample, f(*x*), is calculated as the sum of the baseline value and the SHAP values of all features, and is presented as $f(x)=\;\epsilon [f(x)]+\sum_{i}^{n}\text{SHAP}i$. Where SHAP *i* represents the SHAP value of the *i*-th feature, and *n* denotes the total number of features. The predicted probability (*p*) is computed from *f(x*) utilizing the Sigmoid function: $p=\;\frac{1}{1+e^{-f(x)}}$. In this example, the value of *f(x*) is -1.74, with a predicted probability of 0.15.

## 4 Discussion

There is currently no ML prediction model research focusing on in-hospital death from any cause in ICU-DKD patients. In this research, we created and tested an interpretable ML prediction model utilizing MIMIC-IV and YTU-ICU data banks to explore the risk of death from any cause in ICU-DKD patients. The main findings were as follows: (1) The XGBoost model demonstrated good performance in predicting in-hospital death from any cause across individuals with ICU-DKD, providing strong support for early risk identification and personalized intervention; (2) The model exhibited satisfactory generalizability in the external validation cohort. Despite differences in patient characteristics, the model still demonstrated stable predictive performance, laying the foundation for its application in different clinical environments. (3) SHAP analysis revealed that respiratory failure, lymphocyte count, SOFA score, RDW, and age were key factors affecting the mortality risk, providing an important basis for clinical decision-making.

With the advances in ML, XGBoost has emerged as a highly effective and widely adopted approach for constructing predictive models in healthcare. Prior findings have demonstrated that XGBoost models are highly effective in predicting acute kidney injury in individuals with diabetic ketoacidosis (26). Consequently, XGBoost emerges as a promising tool for the creation of predictive models for medical applications. Predictive modeling for ICU-DKD patients was achieved through the application of ML techniques. Ten most informative variables were selected from an initial set of 45 clinical variables and employed to construct eight ML models, which subsequently underwent internal verification and external validation. Research findings indicated that the XGBoost model proved to be the most effective for predicting in-hospital death from any cause, with an AUC of 0.738 on the internal validation set. The predictive performance of XGBoost in this study is likely attributable to its ensemble learning framework. Specifically, the aggregation of predictions derived from a multitude of decision trees facilitates the accurate representation of intricate non-linear interactions among predictor variables. XGBoost incorporates inherent feature selection capabilities, allowing it to eliminate redundant features and mitigate the susceptibility to overfitting (27). Moreover, it exhibits resilience to missing data and high dimensionality, contributing to its improved performance in clinical prediction (28). To explore the reliability of the XGBoost model, external validation was performed utilizing comprehensive data from the YTU-ICU database. The external validation set achieved an AUROC of 0.746, outperforming the traditional SOFA and SAPS II models. We recognize that the performance of ML algorithms is intrinsically linked to the underlying data distribution, and no single model is universally optimal for all clinical applications. Consequently, we selected XGBoost as the primary model due to its good performance within the specific context and characteristics of our datasets.

Despite this satisfactory discrimination, the model exhibited suboptimal calibration in the external cohort, which may stem from the higher disease severity in the YTU-ICU population. The YTU-ICU population showed elevated SAPS II scores and a higher prevalence of sepsis compared to the training set from MIMIC-IV. Given these differences in baseline risks, the model may systematically underestimate the absolute risk of mortality in the external cohort. Hence, recalibration is necessary for deploying predictive models across heterogeneous healthcare settings.

SHAP was employed to interpret an XGBoost model, focusing on several key variables linked to in-hospital death from any cause across individuals with ICU-DKD. Studies have found that respiratory failure is the primary determinant in forecasting the death risk. Previous studies have indicated that respiratory failure is a critical predictor of adverse outcomes across many critically ill patients, especially those with sepsis, and showed a close correlation with the worsening of the patient’s health and respiratory function decline (29). Respiratory failure enhances neural reflexes in the respiratory center via activation of chemoreceptors and mechanoreceptors in the lungs, leading to an increased respiratory rate and decreased tidal volume. This, in turn, results in a rapid and shallow breathing pattern, further exacerbating the patient’s condition (30). In addition, inflammatory responses and lung injury can lead to a deterioration of pulmonary function and potentially trigger multi-organ failure (31). Our research underscores the pivotal role of respiratory failure in forecasting in-hospital death, particularly in patients with ICU-DKD, where its predictive ability for mortality is most pronounced.

The functional status of the immune system markedly affects the clinical outcomes of individuals within the ICU environment. Particularly among ICU patients with diabetes, immunosuppression and immune dysfunction often exacerbate the patient’s condition and increase the risk of mortality (32). Immunosuppression can be attributed to a multitude of factors, involving diabetes-related immune dysfunction (33), chronic inflammation (34), and medication regimens (35). Given this background, lymphocyte count variations, as a central constituent of the immune system, may be an important predictor of mortality risk. Lymphocytes are principally involved in the recognition and clearance of foreign pathogens, and play a regulatory role in immune responses (36). A decrease in lymphocyte count generally implies suppression or insufficiency of the immune system (37). Patients with DKD exhibit impaired immune function accompanied by chronic low-grade inflammation. This state of immunosuppression predisposes patients to infections, which are recognized as a significant contributor to mortality within the ICU setting (38, 39). Importantly, the immunosuppressed state is not solely characterized by lymphopenia; it can also modulate the balance of inflammatory markers by influencing the overall immune response. For example, the neutrophil-to-lymphocyte ratio (NLR), platelet-to-lymphocyte ratio (PLR), and systemic immune-inflammation index (SII) provide important insights into immune status and inflammatory responses (40). Research involving two cohorts has indicated that an increased NLR is significantly correlated with immune suppression, chronic inflammatory conditions, and the risk of death from any cause in individuals with DKD (41, 42). Consequently, changes in lymphocyte count are an important determinant for evaluating immune dysfunction and predicting mortality risk in ICU-DKD patients.

Existing research has confirmed that SOFA and SAPS II, as comprehensive tools integrating multiple organ function parameters, hold significant clinical importance in the prognostic evaluation of various diseases in the ICU (43). Our findings indicate that higher SOFA scores and SAPS II scores demonstrate a marked correlation with an elevated risk of in-hospital death from any cause among individuals with ICU-DKD. Clinical research focusing on a specific group of critically ill patients has shown that, among kidney transplant recipients on their initial ICU admission, dynamic changes in the early SOFA score are significantly associated with 90-day clinical outcomes (44). Among patients receiving CRRT, the SAPS II scoring system exhibits a significant predictive capacity for the mortality risk within the initial 48 hours following a surgical procedure (45). The application of SOFA and SAPS II scoring systems in the prognostic evaluation of ICU patients offers robust evidence to support early risk stratification and inform targeted interventions. Specifically, the clinical interventions due to risks predicted by the model should be tailored according to local ICU protocols. At lower probability thresholds, physiological monitoring should be enhanced, while early multi-disciplinary consultations should be conducted at higher-risk thresholds.

The pathogenesis of DKD is complex, and current research consistently indicates that inflammation is a key factor in the onset and aggravation of DKD (46). Inflammation may lead to elevated RDW through mechanisms involving the regulation of erythropoiesis, a reduction in red blood cell lifespan, and changes in the homogeneity of red blood cell size (47). RDW, a measure of red blood cell size heterogeneity, provides insight into the degree of oxidative stress and inflammation within an organism (48, 49). Consequently, RDW is considered a potential prognostic factor for risk stratification in patients with DKD. Recent studies have increasingly confirmed the value of RDW in forecasting DKD incidence and clinical outcomes (50–52). Furthermore, treatment-related variables such as vancomycin and meropenem are included in our model, which, however, should be interpreted to reflect the severity of diseases. These antibiotics serve as surrogate markers for the presence of severe or resistant infections rather than as causal contributors to mortality. Their high predictive weight indicates the critical condition of patients who require such potent antimicrobial therapy.

It is worth noting that the final predictors selected in our model, such as SOFA, SAPS II, and respiratory failure, reflect general systemic severity rather than DKD-specific parameters like HbA1c or UACR. This can be attributed to several factors. First, markers for chronic DKD (e.g., HbA1c and proteinuria) often have high missing rates in acute ICU settings. Therefore, these variables were excluded during our initial data preprocessing (missingness > 20%). Second, while indicators like creatinine and BUN were included in the initial LASSO regression, they were superseded by composite scores and acute complication markers. This suggests that for critically ill DKD patients, the immediate risk of in-hospital mortality is attributable more to acute physiological decompensation and multi-organ failure than baseline chronic kidney disease. Our model thus captures the acute-on-chronic characteristic in high-risk ICU-DKD populations.

### 4.1 Strengths and Limitations

Compared to previous studies, this study offers several advantages. Firstly, this research leveraged the MIMIC-IV database, which offers a larger, updated, and higher-quality dataset. Secondly, multiple ML algorithms were employed to assess the predictive capacity of various models. The optimal model was selected based on its overall performance across the evaluation metrics. Furthermore, external validation was performed, which enhanced the model’s accuracy and generalizability and confirmed its applicability across diverse healthcare settings. Finally, SHAP analysis was employed to provide in-depth interpretability of the XGBoost model, revealing key factors influencing mortality risk in patients with DKD and offering valuable insights for clinical decision-making.

However, our research has some limitations. First of all, this research encompassed a variety of clinical characteristics and laboratory indicators. However, due to missing data surpassing 20%, certain hazard factors linked to poor prognosis were excluded from the analysis. Secondly, the mortality risk prediction model is based on data gathered within the first 24 hours of ICU admission, potentially missing later events that might affect outcomes and introducing confounding variables. Furthermore, we acknowledge the presence of clinical collinearity among several top-ranked predictors. Factors such as respiratory failure, pneumonia, and the administration of broad-spectrum antibiotics are clinically intertwined and often reflect a severe infection or sepsis syndrome. Additionally, the SOFA score inherently incorporates parameters related to respiratory dysfunction, which may overlap with respiratory failure. While XGBoost is robust in handling multi-dimensional data, this collinearity may influence the individual SHAP importance assigned to these features. Therefore, the SHAP results should be interpreted as identifying several indicators that collectively reflect a systemic severe infection or sepsis syndrome rather than independent risk factors. Additionally, while an external validation cohort was employed, the limited sample size may restrict the applicability of the XGBoost model in clinical settings. Lastly, although an advanced ML algorithm was employed for prediction, the model employed in research has not yet been developed into an application for direct clinical use. Future research could consider converting our model into an operational online application platform, enabling clinicians and healthcare providers to conveniently use this tool for clinical prediction and management. Furthermore, given the inherent dataset shift between different healthcare centers, local recalibration may be necessary to optimize the predictive performance of the model before clinical deployment in heterogeneous populations.

## 5 Conclusion

We have developed an interpretable XGBoost prediction model that demonstrates good performance in predicting in-hospital death from any cause for patients with severe DKD. SHAP provides an intuitive explanation of importance ranking, threshold values for individual features, and the positive or negative correlations of each feature with outcomes. This aids healthcare professionals in early identification and targeted management, thereby promoting recovery and survival in patients with severe DKD. However, because external calibration was suboptimal, local recalibration and cohort-specific threshold selection should be considered before applying the model in heterogeneous clinical settings.

## Data Availability

The datasets presented in this study can be found in online repositories. The names of the repository/repositories and accession number(s) can be found below: The datasets analyzed during the current study are available from the MIMIC-IV database V3.1.
