# Supplementary material for: Development and external validation of a machine learning model for predicting in-hospital mortality in ICU patients with diabetic kidney disease: a study utilizing the MIMIC database and a Chinese cohort
Source: Front Endocrinol (Lausanne). 2026 Feb 27;17:1699647. doi: 10.3389/fendo.2026.1699647 (PMC12982039; doi:10.3389/fendo.2026.1699647)
Supplement: Supplementary file 1 [file DataSheet1.doc]

**Supplementary Table 1. Summary of Missing Data Rates for Included Variables Across Cohorts**

| **Category** | **Variable** | **MIMIC-IV (n=3,403)** | **YTU-ICU (n=261)** |
| --- | --- | --- | --- |
| **Demographics** | Age | 0 (0.0%) | 0 (0.0%) |
|  | BMI | 387 (11.4%) | 0 (0.0%) |
|  | Gender | 0 (0.0%) | 0 (0.0%) |
|  | Marital status | 0 (0.0%) | 1 (0.38%) |
| **Vital Signs** | Pulse | 1 (0.03%) | 0 (0.0%) |
|  | Respiratory rate | 1 (0.03%) | 0 (0.0%) |
| **Laboratory Indicators** | Albumin | 484 (14.2%) | 1 (0.38%) |
|  | ALT | 322 (9.5%) | 0 (0.0%) |
|  | AST | 241 (7.1%) | 0 (0.0%) |
|  | APTT | 94 (2.8%) | 0 (0.0%) |
|  | Bicarbonate | 4 (0.1%) | 0 (0.0%) |
|  | BUN | 6 (0.2%) | 0 (0.0%) |
|  | Chloride | 5 (0.1%) | 0 (0.0%) |
|  | Creatinine | 4 (0.1%) | 0 (0.0%) |
|  | Glucose | 4 (0.1%) | 0 (0.0%) |
|  | Hemoglobin | 17 (0.5%) | 0 (0.0%) |
|  | Lymphocyte count | 301 (8.8%) | 1 (0.38%) |
|  | Neutrophils | 299 (8.8%) | 0 (0.0%) |
|  | Platelet count | 22 (0.6%) | 0 (0.0%) |
|  | Potassium | 5 (0.1%) | 0 (0.0%) |
|  | Prothrombin time (PT) | 83 (2.4%) | 0 (0.0%) |
|  | Red cell distribution width (RDW) | 22 (0.6%) | 0 (0.0%) |
|  | Sodium | 5 (0.1%) | 0 (0.0%) |
|  | Total bilirubin | 322 (9.5%) | 0 (0.0%) |
|  | Leucocyte count | 19 (0.6%) | 0 (0.0%) |
| **Severity Scores** | SAPS II score | 0 (0.0%) | 0 (0.0%) |
|  | SOFA score | 0 (0.0%) | 0 (0.0%) |
|  | GCS score | 3 (0.1%) | 0 (0.0%) |
| **Comorbidities** | All comorbidities* | 0 (0.0%) | 0 (0.0%) |

*Note: In the final analysis cohort, patients with missing data in essential categorical variables (including comorbidities and medication history) were excluded during the initial data cleaning phase.

**Supplementary Table 2. Model Training and Hyperparameter Specifications**

| **Model** | **R Package** | **Tuning Strategy** | **Main Hyperparameters(Final Settings/Ranges)** |
| --- | --- | --- | --- |
| XGBoost | xgboost | Grid Search | max_depth (3), eta (0.01), nrounds (200), gamma (0.2), min_child_weight (10) |
| GBDT | gbm | Grid Search | n.trees (100), interaction.depth (5), shrinkage (0.01), n.minobsinnode (10) |
| RF | randomForest | Grid Search | ntree (300), mtry (2), nodesize (30) |
| SVM | e1071 | Grid Search | Kernel: Radial, cost (10), gamma (0.0625) |
| NN | nnet | Grid Search | size (5-15), decay (0.001-0.1) |
| NB | e1071 | Fixed | Default settings |
| LR | stats | Fixed | Binomial family, Logit link function |

**Supplementary Table 3.** Performance of machine learning models.

| **Algorithm** | **Accuracy (%)** | **Best cutoff** | **Youden index (%)** | **Sensitivit y(%)** | **Specificity (%)** | **F1 score** | **Recall (%)** | **PPV (%)** | **NPV (%)** |
| --- | --- | --- | --- | --- | --- | --- | --- | --- | --- |
| Training set |  |  |  |  |  |  |  |  |  |
| XGBoost | 67.17 | 0.224 | 47.52 | 84.14 | 63.38 | 0.483 | 84.14 | 33.92 | 94.7 |
| GBDT | 67.38 | 0.153 | 48.67 | 85.29 | 63.38 | 0.488 | 85.29 | 34.23 | 95.07 |
| LightGBM | 70.19 | 0.193 | 44.79 | 75.86 | 68.92 | 0.482 | 75.86 | 35.29 | 92.74 |
| NN | 66.12 | 0.136 | 34.99 | 69.66 | 65.33 | 0.429 | 69.66 | 30.98 | 90.6 |
| LR | 66.92 | 0.153 | 43.64 | 79.54 | 64.1 | 0.468 | 79.54 | 33.11 | 93.34 |
| NB | 67.46 | 0.128 | 41.27 | 75.63 | 65.64 | 0.459 | 75.63 | 32.97 | 92.34 |
| RF | 80.52 | 0.105 | 63.49 | 83.68 | 79.82 | 0.611 | 83.68 | 48.08 | 95.63 |
| SVM | 83.33 | 0.167 | 58.54 | 72.87 | 85.67 | 0.615 | 72.87 | 53.18 | 93.39 |
| Internal validation set |  |  |  |  |  |  |  |  |  |
| XGBoost | 72.18 | 0.26 | 37.83 | 63.78 | 74.04 | 0.454 | 63.78 | 35.22 | 90.23 |
| GBDT | 64.45 | 0.153 | 36.37 | 74.05 | 62.32 | 0.43 | 74.05 | 30.31 | 91.56 |
| LightGBM | 73.07 | 0.21 | 35.54 | 59.46 | 76.08 | 0.444 | 59.46 | 35.48 | 89.45 |
| NN | 60.03 | 0.113 | 27.62 | 69.73 | 57.89 | 0.387 | 69.73 | 26.82 | 89.63 |
| LR | 71.01 | 0.192 | 36.39 | 63.78 | 72.61 | 0.444 | 63.78 | 34.01 | 90.06 |
| NB | 70.52 | 0.18 | 34.53 | 62.16 | 72.37 | 0.433 | 62.16 | 33.24 | 89.63 |
| RF | 69.15 | 0.095 | 32.86 | 62.16 | 70.69 | 0.422 | 62.16 | 31.94 | 89.41 |
| SVM | 71.89 | 0.168 | 31.58 | 56.22 | 75.36 | 0.42 | 56.21 | 33.54 | 88.61 |

Abbreviation: XGBoost, eXtreme Gradient Boosting; GBDT, Gradient Boosting Decision Tree; LightGBM, Light Gradient Boosting Machine; NN, Neural Network; LR, Logistic Regression; NB, Naive Bayes; RF, Random Forest; SVM, Support Vector Machine; PPV, positive predictive value; NPV, negative predictive value.

**Supplementary Table 4. Discrimination and calibration performance across models and cohorts.**

| **Cohort** | **Model** | **AUROC** | **PR-AUC** | **Brier** | **Calibration intercept** | **Calibration slope** |
| --- | --- | --- | --- | --- | --- | --- |
| Train | GBDT | 0.811 (0.789–0.831) | 0.508 (0.459–0.554) | 0.122 (0.114–0.131) | 0.006 (-0.097–0.110) | 1.729 (1.568–1.906) |
| Train | LR | 0.775 (0.751–0.797) | 0.426 (0.380–0.475) | 0.127 (0.119–0.137) | 0.000 (-0.116–0.115) | 1.000 (0.894–1.114) |
| Train | LightGBM | 0.799 (0.777–0.820) | 0.496 (0.449–0.542) | 0.129 (0.121–0.138) | -0.001 (-0.102–0.102) | 2.455 (2.209–2.716) |
| Train | NB | 0.766 (0.742–0.787) | 0.406 (0.360–0.451) | 0.151 (0.141–0.162) | -0.391 (-0.587–-0.221) | 0.435 (0.384–0.490) |
| Train | NN | 0.795 (0.773–0.816) | 0.448 (0.398–0.496) | 0.124 (0.116–0.132) | -0.010 (-0.127–0.103) | 1.056 (0.934–1.191) |
| Train | RF | 0.904 (0.890–0.917) | 0.697 (0.652–0.738) | 0.108 (0.099–0.118) | 0.895 (-2907865754363907–0.983) | 1.355 (1.231–1.495) |
| Train | SVM | 0.819 (0.794–0.841) | 0.589 (0.539–0.633) | 0.126 (0.118–0.136) | 0.000 (-0.103–0.103) | 2.784 (2.221–3.509) |
| Train | XGBoost | 0.810 (0.790–0.830) | 0.497 (0.449–0.544) | 0.127 (0.120–0.134) | -0.285 (-0.388–-0.183) | 2.286 (2.069–2.520) |
| Internal | GBDT | 0.733 (0.691–0.770) | 0.402 (0.334–0.476) | 0.131 (0.118–0.146) | 0.046 (-0.116–0.207) | 1.262 (1.023–1.512) |
| Internal | LR | 0.728 (0.686–0.765) | 0.379 (0.312–0.448) | 0.133 (0.119–0.147) | 0.059 (-0.116–0.238) | 0.845 (0.671–1.020) |
| Internal | LightGBM | 0.731 (0.690–0.768) | 0.413 (0.344–0.485) | 0.135 (0.121–0.149) | 0.021 (-0.139–0.179) | 1.861 (1.518–2.220) |
| Internal | NB | 0.723 (0.682–0.759) | 0.368 (0.301–0.438) | 0.155 (0.139–0.172) | -0.224 (-0.502–0.038) | 0.397 (0.316–0.476) |
| Internal | NN | 0.730 (0.687–0.767) | 0.387 (0.319–0.456) | 0.133 (0.119–0.147) | 0.057 (-0.122–0.239) | 0.707 (0.550–0.880) |
| Internal | RF | 0.722 (0.682–0.760) | 0.383 (0.315–0.456) | 0.140 (0.124–0.159) | 0.909 (0.701–1.114) | 0.132 (0.055–0.470) |
| Internal | SVM | 0.670 (0.623–0.715) | 0.357 (0.292–0.425) | 0.140 (0.125–0.156) | 0.027 (-0.136–0.185) | 1.253 (0.883–1.758) |
| Internal | XGBoost | 0.738 (0.697–0.775) | 0.411 (0.342–0.486) | 0.134 (0.122–0.146) | -0.267 (-0.428–-0.109) | 1.667 (1.347–2.000) |
| External | GBDT | 0.746 (0.688–0.805) | 0.834 (0.775–0.886) | 0.387 (0.348–0.424) | 2.082 (1.820–2.344) | 1.639 (1.205–2.211) |
| External | LR | 0.761 (0.701–0.821) | 0.844 (0.787–0.895) | 0.379 (0.339–0.418) | 2.240 (1.971–2.514) | 1.148 (0.824–1.589) |
| External | LightGBM | 0.735 (0.674–0.795) | 0.828 (0.768–0.881) | 0.398 (0.361–0.435) | 2.021 (1.773–2.279) | 2.232 (1.630–3.016) |
| External | NB | 0.713 (0.650–0.773) | 0.816 (0.755–0.869) | 0.364 (0.320–0.407) | 2.579 (2.200–2.946) | 0.413 (0.264–0.617) |
| External | NN | 0.702 (0.636–0.767) | 0.814 (0.753–0.867) | 0.397 (0.354–0.438) | 2.473 (2.141–2.788) | 0.591 (0.389–0.859) |
| External | RF | 0.712 (0.647–0.772) | 0.819 (0.761–0.870) | 0.447 (0.402–0.494) | 2.945 (2.610–3.297) | 0.188 (0.053–0.764) |
| External | SVM | 0.560 (0.490–0.628) | 0.714 (0.639–0.782) | 0.418 (0.378–0.458) | 2.085 (1.830–2.358) | 0.740 (0.322–1.341) |
| External | XGBoost | 0.746 (0.686–0.805) | 0.834 (0.774–0.887) | 0.357 (0.324–0.389) | 1.783 (1.532–2.043) | 1.954 (1.422–2.647) |

**Supplementary Table 5.** Net benefit across threshold probabilities (0.1–0.5) for all models and cohorts.

| **Cohort** | **Model** | **NB@0.1** | **NB@0.2** | **NB@0.3** | **NB@0.4** | **NB@0.5** |
| --- | --- | --- | --- | --- | --- | --- |
| Train | GBDT | 0.118 (0.102–0.135) | 0.078 (0.063–0.092) | 0.045 (0.034–0.056) | 0.025 (0.017–0.033) | 0.011 (0.005–0.016) |
| Train | LR | 0.114 (0.099–0.130) | 0.067 (0.052–0.081) | 0.032 (0.020–0.044) | 0.013 (0.002–0.024) | 0.008 (0.000–0.017) |
| Train | LightGBM | 0.092 (0.075–0.110) | 0.072 (0.058–0.087) | 0.030 (0.021–0.038) | 0.010 (0.006–0.015) | 0.002 (0.000–0.004) |
| Train | NB | 0.108 (0.093–0.123) | 0.065 (0.051–0.081) | 0.023 (0.009–0.037) | -0.003 (-0.018–0.011) | -0.024 (-0.040–-0.008) |
| Train | NN | 0.120 (0.104–0.135) | 0.077 (0.062–0.092) | 0.036 (0.022–0.050) | 0.024 (0.012–0.035) | 0.008 (0.000–0.016) |
| Train | RF | 0.135 (0.121–0.151) | 0.097 (0.085–0.111) | 0.066 (0.056–0.078) | 0.042 (0.033–0.051) | 0.019 (0.013–0.025) |
| Train | SVM | 0.093 (0.077–0.111) | 0.069 (0.059–0.080) | 0.033 (0.025–0.041) | 0.024 (0.017–0.031) | 0.019 (0.013–0.026) |
| Train | XGBoost | 0.094 (0.077–0.112) | 0.073 (0.056–0.089) | 0.051 (0.037–0.065) | 0.024 (0.015–0.032) | 0.008 (0.003–0.012) |
| Internal | GBDT | 0.106 (0.081–0.131) | 0.057 (0.036–0.079) | 0.022 (0.007–0.037) | 0.020 (0.008–0.031) | 0.009 (0.001–0.017) |
| Internal | LR | 0.098 (0.075–0.123) | 0.059 (0.037–0.081) | 0.028 (0.011–0.046) | 0.013 (-0.002–0.027) | -0.002 (-0.015–0.010) |
| Internal | LightGBM | 0.090 (0.065–0.117) | 0.054 (0.032–0.076) | 0.024 (0.013–0.037) | 0.008 (0.002–0.015) | 0.001 (0.000–0.003) |
| Internal | NB | 0.092 (0.071–0.115) | 0.056 (0.034–0.077) | 0.019 (-0.001–0.040) | -0.005 (-0.025–0.016) | -0.028 (-0.051–-0.007) |
| Internal | NN | 0.099 (0.076–0.123) | 0.056 (0.034–0.078) | 0.029 (0.009–0.049) | 0.016 (0.001–0.031) | 0.003 (-0.009–0.015) |
| Internal | RF | 0.085 (0.066–0.105) | 0.039 (0.024–0.056) | 0.019 (0.006–0.032) | 0.013 (0.003–0.023) | 0.006 (-0.002–0.014) |
| Internal | SVM | 0.091 (0.065–0.118) | 0.036 (0.022–0.050) | 0.014 (0.005–0.024) | 0.007 (-0.001–0.015) | 0.004 (-0.004–0.012) |
| Internal | XGBoost | 0.090 (0.064–0.118) | 0.048 (0.023–0.074) | 0.026 (0.007–0.045) | 0.018 (0.006–0.030) | 0.004 (-0.002–0.010) |
| External | GBDT | 0.553 (0.488–0.618) | 0.336 (0.275–0.395) | 0.128 (0.086–0.174) | 0.058 (0.031–0.088) | 0.035 (0.015–0.058) |
| External | LR | 0.524 (0.462–0.589) | 0.312 (0.250–0.370) | 0.169 (0.120–0.219) | 0.094 (0.058–0.131) | 0.058 (0.031–0.088) |
| External | LightGBM | 0.590 (0.526–0.654) | 0.362 (0.299–0.425) | 0.069 (0.038–0.104) | 0.019 (0.004–0.038) | 0.004 (0.000–0.012) |
| External | NB | 0.419 (0.356–0.479) | 0.291 (0.230–0.353) | 0.227 (0.171–0.282) | 0.159 (0.108–0.212) | 0.142 (0.100–0.188) |
| External | NN | 0.472 (0.408–0.537) | 0.300 (0.241–0.357) | 0.185 (0.136–0.237) | 0.112 (0.073–0.154) | 0.062 (0.035–0.092) |
| External | RF | 0.411 (0.348–0.471) | 0.187 (0.140–0.237) | 0.088 (0.054–0.123) | 0.038 (0.015–0.065) | 0.027 (0.008–0.046) |
| External | SVM | 0.575 (0.508–0.643) | 0.133 (0.088–0.178) | 0.054 (0.027–0.085) | 0.054 (0.027–0.085) | 0.038 (0.015–0.065) |
| External | XGBoost | 0.591 (0.526–0.656) | 0.434 (0.367–0.497) | 0.202 (0.152–0.256) | 0.054 (0.027–0.085) | 0.019 (0.004–0.038) |

**Supplementary Table 6.** All predictor variables for critically ill patients with DKD in the YTU-ICU database collection.

|  | **Overall (N=260)** | **Alive (N=96)** | **Death (N=164)** | **P value** |
| --- | --- | --- | --- | --- |
| Demographics |  |  |  |  |
| Gender: |  |  |  | 0.657 |
| Male | 171 (65.8%) | 61 (63.5%) | 110 (67.1%) |  |
| Female | 89 (34.2%) | 35 (36.5%) | 54 (32.9%) |  |
| Age | 71.0 [65.0;78.0] | 66.0 [57.0;73.0] | 73.0 [68.0;81.0] | <0.001 |
| Marital status: |  |  |  | 0.28 |
| Single | 9 (3.46%) | 5 (5.21%) | 4 (2.44%) |  |
| Divorced/Widowed | 33 (12.7%) | 9 (9.38%) | 24 (14.6%) |  |
| Married | 218 (83.8%) | 82 (85.4%) | 136 (82.9%) |  |
| BMI | 22.1 [20.3;23.4] | 21.5 [19.6;22.9] | 22.4 [21.1;23.6] | 0.002 |
| Vital signs |  |  |  |  |
| DBP | 76.4 (17.8) | 73.3 (19.3) | 78.2 (16.6) | 0.038 |
| SBP | 132 (30.7) | 128 (29.9) | 134 (31.0) | 0.129 |
| Pulse | 90.0 [80.0;102] | 88.0 [79.8;101] | 90.0 [81.0;104] | 0.319 |
| Respiratory | 20.0 [19.0;21.2] | 20.0 [19.0;21.0] | 20.0 [19.0;22.0] | 0.655 |
| Respiratory | 20.0 [19.0;21.2] | 20.0 [19.0;21.0] | 20.0 [19.0;22.0] | 0.655 |
| Laboratory indicators |  |  |  |  |
| ALB | 33.8 [30.4;37.5] | 33.7 [29.7;37.1] | 34.0 [30.8;37.8] | 0.452 |
| ALT | 20.0 [10.0;39.0] | 19.0 [9.00;39.0] | 20.5 [10.0;38.8] | 0.922 |
| APTT | 30.7 [26.5;36.5] | 29.1 [26.1;34.0] | 31.6 [27.0;37.5] | 0.062 |
| AST | 27.0 [16.0;48.0] | 20.5 [13.8;47.2] | 30.0 [18.0;50.0] | 0.007 |
| BIC | 22.8 [19.8;25.3] | 22.4 [19.8;25.1] | 23.0 [20.2;25.4] | 0.578 |
| BUN | 34.2 [22.3;49.3] | 30.1 [22.2;47.1] | 35.6 [22.3;50.7] | 0.443 |
| CL | 103 [99.1;107] | 104 [99.4;107] | 102 [98.5;107] | 0.631 |
| CR | 179 [120;301] | 177 [118;304] | 183 [122;300] | 0.984 |
| GLU | 12.4 [9.11;18.0] | 11.2 [8.17;17.0] | 12.8 [9.88;18.3] | 0.055 |
| HB | 109 (26.9) | 107 (27.6) | 110 (26.5) | 0.466 |
| LYM | 0.77 [0.51;1.14] | 0.91 [0.53;1.30] | 0.72 [0.50;1.08] | 0.044 |
| NEU | 8.94 [6.31;13.0] | 8.95 [6.11;14.6] | 8.94 [6.37;12.6] | 0.654 |
| PLT | 174 [123;236] | 184 [123;245] | 168 [125;227] | 0.398 |
| K | 4.30 [3.86;5.08] | 4.28 [3.86;5.06] | 4.38 [3.87;5.16] | 0.826 |
| PT | 13.2 [11.9;14.8] | 13.5 [12.4;14.7] | 13.0 [11.6;14.9] | 0.134 |
| RDW | 13.9 [13.0;15.2] | 13.7 [12.9;15.3] | 13.9 [13.1;15.1] | 0.344 |
| NA | 138 [134;142] | 138 [134;141] | 138 [133;142] | 0.851 |
| TB | 10.8 [6.10;18.5] | 9.00 [5.30;14.0] | 11.7 [6.83;20.2] | 0.009 |
| LEU | 10.8 [7.68;15.2] | 10.7 [7.55;16.3] | 10.9 [7.82;14.2] | 0.682 |
| Comorbidity disease |  |  |  |  |
| Acidosis: |  |  |  | 0.148 |
| No | 206 (79.2%) | 71 (74.0%) | 135 (82.3%) |  |
| Yes | 54 (20.8%) | 25 (26.0%) | 29 (17.7%) |  |
| Arrhythmia: |  |  |  | 0.005 |
| No | 123 (47.3%) | 34 (35.4%) | 89 (54.3%) |  |
| Yes | 137 (52.7%) | 62 (64.6%) | 75 (45.7%) |  |
| CVD: |  |  |  | 0.002 |
| No | 151 (58.1%) | 68 (70.8%) | 83 (50.6%) |  |
| Yes | 109 (41.9%) | 28 (29.2%) | 81 (49.4%) |  |
| Electrolyte disturbance: |  |  |  | 0.297 |
| No | 153 (58.8%) | 52 (54.2%) | 101 (61.6%) |  |
| Yes | 107 (41.2%) | 44 (45.8%) | 63 (38.4%) |  |
| Pneumonia: |  |  |  | 0.312 |
| No | 102 (39.2%) | 42 (43.8%) | 60 (36.6%) |  |
| Yes | 158 (60.8%) | 54 (56.2%) | 104 (63.4%) |  |
| Respiratory failure: |  |  |  | <0.001 |
| No | 127 (48.8%) | 66 (68.8%) | 61 (37.2%) |  |
| Yes | 133 (51.2%) | 30 (31.2%) | 103 (62.8%) |  |
| Sepsis: |  |  |  | <0.001 |
| No | 236 (90.8%) | 96 (100%) | 140 (85.4%) |  |
| Yes | 24 (9.23%) | 0 (0.00%) | 24 (14.6%) |  |
| Medication history |  |  |  |  |
| Cephalosporin: |  |  |  | 0.691 |
| No | 138 (53.1%) | 53 (55.2%) | 85 (51.8%) |  |
| Yes | 122 (46.9%) | 43 (44.8%) | 79 (48.2%) |  |
| Dexamethasone: |  |  |  | <0.001 |
| No | 230 (88.5%) | 96 (100%) | 134 (81.7%) |  |
| Yes | 30 (11.5%) | 0 (0.00%) | 30 (18.3%) |  |
| Insulin: |  |  |  | 0.001 |
| No | 100 (38.5%) | 24 (25.0%) | 76 (46.3%) |  |
| Yes | 160 (61.5%) | 72 (75.0%) | 88 (53.7%) |  |
| Meropenem: |  |  |  | 0.274 |
| No | 147 (56.5%) | 59 (61.5%) | 88 (53.7%) |  |
| Yes | 113 (43.5%) | 37 (38.5%) | 76 (46.3%) |  |
| Omeprazole: |  |  |  | 1 |
| No | 202 (77.7%) | 75 (78.1%) | 127 (77.4%) |  |
| Yes | 58 (22.3%) | 21 (21.9%) | 37 (22.6%) |  |
| Vancomycin: |  |  |  | 0.129 |
| No | 246 (94.6%) | 94 (97.9%) | 152 (92.7%) |  |
| Yes | 14 (5.38%) | 2 (2.08%) | 12 (7.32%) |  |
| Medical operation |  |  |  |  |
| CRRT: |  |  |  | 0.004 |
| No | 248 (95.4%) | 96 (100%) | 152 (92.7%) |  |
| Yes | 12 (4.62%) | 0 (0.00%) | 12 (7.32%) |  |
| MV: |  |  |  | 0.552 |
| No | 117 (45.0%) | 46 (47.9%) | 71 (43.3%) |  |
| Yes | 143 (55.0%) | 50 (52.1%) | 93 (56.7%) |  |
| Medical scores |  |  |  |  |
| GCS | 15.0 [15.0;15.0] | 15.0 [15.0;15.0] | 15.0 [15.0;15.0] | 0.005 |
| SAPSII | 39.0 [31.0;48.0] | 37.0 [31.0;44.5] | 41.0 [31.0;50.0] | 0.033 |
| SOFA | 5.00 [3.00;8.00] | 4.00 [2.00;6.00] | 6.00 [4.00;10.0] | <0.001 |

Abbreviation: BMI, Body Mass Index; DBP, Diastolic Blood Pressure; SBP, Systolic Blood Pressure; Alb, Albumin; ALT, Alanine Aminotransferase; APTT, Activated Partial Thromboplastin Time; AST, Aspartate Aminotransferase; Bic, Bicarbonate; BUN, Blood Urea Nitrogen; CL, Chloride; Cr, Creatinine; GLU, Glucose; HB, Hemoglobin; Lym, Lymphocyte; NEU, Neutrophile; PLT, Platelet; K, Potassium; PT, Prothrombin Time; RDW, Red Cell Distribution Width; NA, Sodium; TB, Total Billrubin; LEU, Leukocyte; CVD, Cardiovascular Disease; CRRT, Continuous Renal Replacement Therapy; MV, Mechanical Ventilation; GCS, Glasgow Coma Scale; SAPSII, Simplified Acute Physiology Score II; SOFA, Sequential Organ Failure Assessment.

**Supplementary Table 7.** Performance of machine learning models on external validation set

| **Algorithm** | **Accuracy (%)** | **Best cutoff** | **Youden index (%)** | **Sensitivity (%)** | **Specificity (%)** | **F1 score** | **Recall (%)** | **PPV (%)** | **NPV (%)** |
| --- | --- | --- | --- | --- | --- | --- | --- | --- | --- |
| External validation set |  |  |  |  |  |  |  |  |  |
| XGBoost | 72.69 | 0.216 | 45.04 | 73.17 | 71.88 | 0.772 | 73.17 | 81.63 | 61.06 |
| GBDT | 71.54 | 0.166 | 43.65 | 70.73 | 72.92 | 0.758 | 70.73 | 81.69 | 59.32 |
| LightGBM | 70.38 | 0.179 | 40.96 | 70.12 | 70.83 | 0.749 | 70.12 | 80.42 | 58.12 |
| NN | 65.77 | 0.145 | 31.91 | 65.24 | 66.67 | 0.706 | 65.24 | 76.98 | 52.89 |
| LR | 73.08 | 0.133 | 43.07 | 77.44 | 65.63 | 0.784 | 77.43 | 79.38 | 63 |
| NB | 69.62 | 0.087 | 34.98 | 75.61 | 59.38 | 0.758 | 75.61 | 76.07 | 58.76 |
| RF | 69.23 | 0.068 | 30.48 | 80.49 | 50 | 0.767 | 80.49 | 73.33 | 60 |
| SVM | 52.31 | 0.171 | 12.73 | 40.85 | 71.88 | 0.519 | 40.85 | 71.28 | 41.57 |

Abbreviation: XGBoost, eXtreme Gradient Boosting; GBDT, Gradient Boosting Decision Tree; LightGBM, Light Gradient Boosting Machine; NN, Neural Network; LR, Logistic Regression; NB, Naive Bayes; RF, Random Forest; SVM, Support Vector Machine; PPV, positive predictive value; NPV, negative predictive value.

**
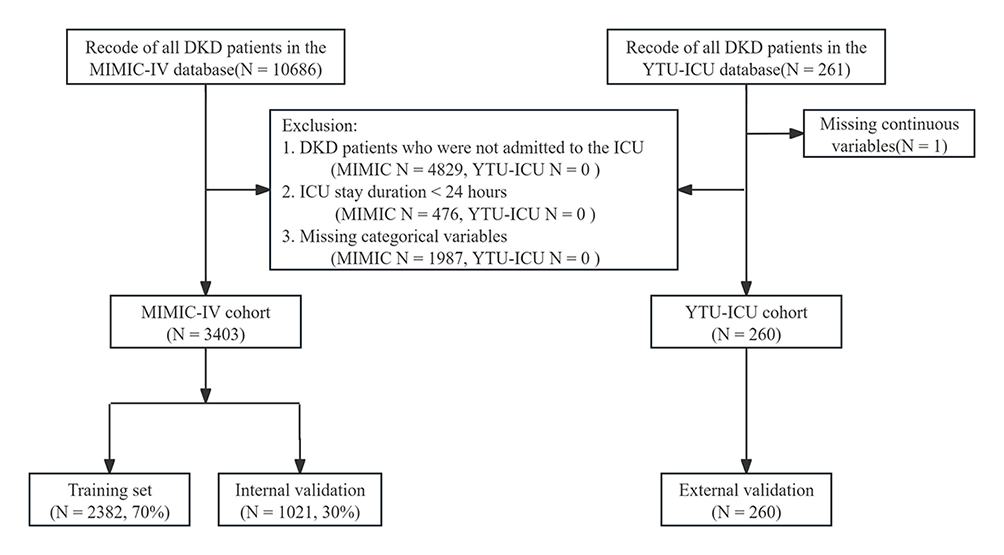
**

**Supplementary Figure 1.** Flowchart of screening

**
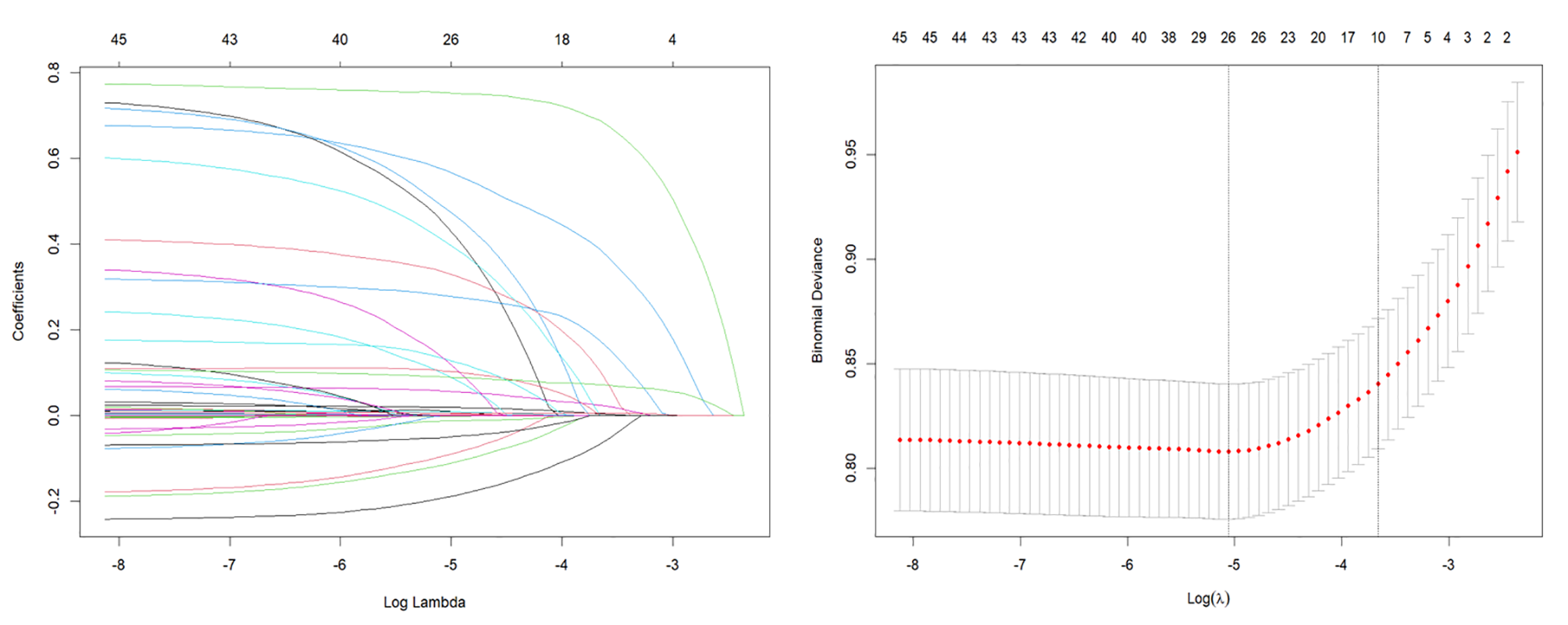
**

**Supplementary Figure 2.** Regularization path and cross-validation results for the Lasso regression model.


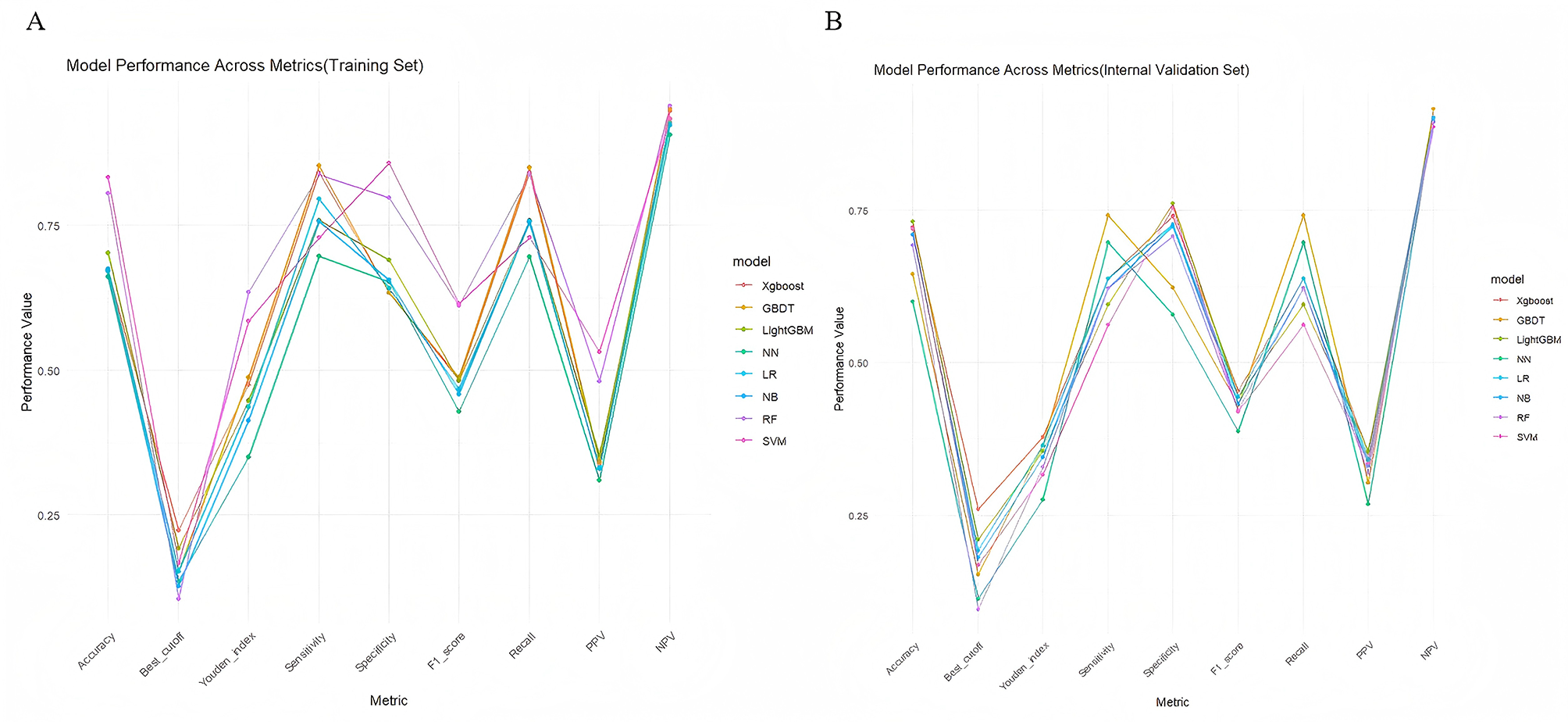


**Supplementary Figure 3.** Line plot illustrating the predictive performance of the eight models.
